# Scaling, Leakage Current Suppression, and Simulation of Carbon Nanotube Field-Effect Transistors

**DOI:** 10.3390/nano15151168

**Published:** 2025-07-28

**Authors:** Weixu Gong, Zhengyang Cai, Shengcheng Geng, Zhi Gan, Junqiao Li, Tian Qiang, Yanfeng Jiang, Mengye Cai

**Affiliations:** School of Integrated Circuits, Jiangnan University, Wuxi 214122, China; 6231916025@stu.jiangnan.edu.cn (W.G.); 6231916024@stu.jiangnan.edu.cn (S.G.); 6231916021@stu.jiangnan.edu.cn (Z.G.); 6231916035@stu.jiangnan.edu.cn (J.L.); qtknight@jiangnan.edu.cn (T.Q.); jiangyf@jiangnan.edu.cn (Y.J.)

**Keywords:** carbon nanotube field-effect transistor, leakage current, TCAD simulation, vertically stacked structure, sidewall gate structure

## Abstract

Carbon nanotube field-effect transistors (CNTFETs) are becoming a strong competitor for the next generation of high-performance, energy-efficient integrated circuits due to their near-ballistic carrier transport characteristics and excellent suppression of short-channel effects. However, CNT FETs with large diameters and small band gaps exhibit obvious bipolarity, and gate-induced drain leakage (GIDL) contributes significantly to the off-state leakage current. Although the asymmetric gate strategy and feedback gate (FBG) structures proposed so far have shown the potential to suppress CNT FET leakage currents, the devices still lack scalability. Based on the analysis of the conduction mechanism of existing self-aligned gate structures, this study innovatively proposed a design strategy to extend the length of the source–drain epitaxial region (L_ext_) under a vertically stacked architecture. While maintaining a high drive current, this structure effectively suppresses the quantum tunneling effect on the drain side, thereby reducing the off-state leakage current (I_off_ = 10^−10^ A), and has good scaling characteristics and leakage current suppression characteristics between gate lengths of 200 nm and 25 nm. For the sidewall gate architecture, this work also uses single-walled carbon nanotubes (SWCNTs) as the channel material and uses metal source and drain electrodes with good work function matching to achieve low-resistance ohmic contact. This solution has significant advantages in structural adjustability and contact quality and can significantly reduce the off-state current (I_off_ = 10^−14^ A). At the same time, it can solve the problem of off-state current suppression failure when the gate length of the vertical stacking structure is 10 nm (the total channel length is 30 nm) and has good scalability.

## 1. Introduction

Since the advent of silicon CMOS technology, the progression of integrated circuits has largely followed the scaling trend articulated by Moore’s Law, which forecasts a twofold increase in transistor integration density on one chip approximately every 18 to 24 months. Integrated circuits have therefore progressed in the direction of smaller channel sizes, higher integration, various functions, and lower power consumption. As device size decreases, some new structures and materials were proposed to solve the bottleneck problems of weakened device gate control ability and large leakage current in short channel conditions, such as strained silicon technology, high-K gate dielectrics, and metal gate technology, and fin transistor structures (FinFET). However, it is about to reach the physical limit of silicon-based CMOS scaling [1,2]: as the gate dimension is reduced to sub-20-nanometer regimes, Si FETs begin to lose control of the channel current. As the size of transistors decreases below 22 nm, the short-channel effect becomes more serious. Before this scale, high-permittivity gate insulators or ultrathin channel engineering have traditionally been employed to enhance electrostatic gate modulation; however, such approaches have proven insufficient here.

Among a range of materials that have the potential to surpass CMOS, carbon nanotubes stand out: carbon nanotube consists of a rolled-up graphene sheet forming a cylindrical nanostructure, where its electronic characteristics are governed by both the tube diameter and the chiral angle. These structural features are quantitatively described by the chiral vector, which defines the tube’s geometry and associated electronic behavior [3,4,5,6,7]. CNTFETs are widely used in the biomedical field due to their high sensitivity and biocompatibility. Research on CNTFET biosensors with junction-free, gate-all-around (GAA) and dual-gate materials has been carried out and achieved good results [8]. At the same time, CNT tunneling field-effect transistors have been studied in implantable medical devices [9], and the results show that TFETs are suitable for energy-constrained long-term operation devices. Yi et al. [10] demonstrated a new ammonia sensor based on multi-walled carbon nanofiber (MWCNF) FET. The study showed that applying a positive gate voltage significantly improved the reversibility and repeatability of the sensor, highlighting the advantages of gate-controlled carbon nanomaterial systems in sensitive detection tasks. A defining characteristic of CNTFETs is the utilization of an SWCNT as the semiconducting channel positioned between two metallic contacts that function as the source and drain electrodes. This structural configuration bears resemblance to that of conventional MOSFETs [11]. There are many advantages of carbon nanotubes as channels: (1) carbon nanotubes exhibit exceptional carrier transport characteristics at ambient temperature, including remarkably high charge carrier mobility and elevated saturation drift velocity; (2) the diameter of carbon nanotubes is less than 3 nm [12], which can better overcome the drain-induced barrier lowering effect (DIBL); (3) carbon nanotubes have an ultra-long mean free path [13], and the one-dimensional structure greatly reduces the phase space of scattering, and the scattering probability is low. The effect of humidity on the performance of carbon nanotubes at room temperature has been studied. At a frequency of 50 kHz, the capacitance of the vertical MWCNT array increased by six times within the same humidity range [14]. Due to the electron donation characteristics of water molecules, in the open channel state (negative gate voltage), humidity pulses cause the source–drain current to decrease; conversely, under positive gate voltage, the source–drain current increases [15]. Some people have studied the use of electron donors doped in growing nanotubes in CNTFETs to make CNTFETs unipolar p-type devices [16]. However, the doped carbon nanotubes destroyed the original periodic potential field, resulting in low mobility. Studies have shown that palladium (Pd) and scandium (Sc) are commonly utilized as contact materials for the source and drain electrodes to enable the fabrication of p-type and n-type CNTFETs, respectively, by selectively facilitating hole or electron injections into the carbon nanotube channel, thereby achieving ohmic contact characteristics [17,18]. Investigations by IBM’s Phaedon group [19,20,21] revealed that the Fermi level of carbon nanotubes can be modulated via gate bias, enabling control over the device current by altering the height and width of the contact Schottky barrier. Notably, the threshold voltage of self-aligned n-type CNTFETs can be tuned through the choice of gate electrode material with varying work functions. Ali et al. proposed a self-aligned CNTFET with a 40 nm channel length, achieving an impressive subthreshold swing as low as 70 mV/decade [22]. Furthermore, fabrication of short-channel, high-performance top-gate CNTFETs has been reported [23,24,25,26], exhibiting superior electrical performance relative to CMOS transistors of equivalent dimensions. Nevertheless, a critical limitation of Schottky barrier (SB) CNTFETs is the electron tunneling through the drain-side Schottky barrier under high gate-to-drain voltage conditions, which induces a significant bias-dependent off-state leakage current, commonly referred to as GIDL [27,28,29]. This leakage current is markedly exacerbated in short-channel CNTFETs during off-state operation [18,26,30]. For applications in high-performance (HP) and standard-performance (SP) domains, leakage current specifications typically mandate levels below 100 nA/µm and 10 nA/µm, respectively. The elevated GIDL-induced leakage current thus poses a substantial challenge for CNTFETs in satisfying the stringent leakage requirements of SP devices. There have been some studies on suppressing GIDL, such as the feedback gate structure [29], asymmetric gate strategy [31], and dual-gate design [32]. However, the above studies have the following characteristics: the structure with a high leakage current suppression function lacks scalability, and the scalable structure often cannot suppress the off-state current. Therefore, it is necessary to study a structure that has both leakage current suppression and scalability.

In this work, we first developed an experimental data calibration model for CNT FET based on experimental results and TCAD. Secondly, by analyzing the cause of the off-state leakage current of this structure, we innovatively proposed a design strategy to extend the length of the source and drain epitaxial region (L_ext_) in the vertical stacking structure. This enables the device to have good scaling characteristics and the ability to suppress the off-state current when L_g_ is between 200 nm and 25 nm. In view of the failure of the vertical stacking structure to suppress the leakage current in a short channel (L_g_ = 10 nm), we innovatively proposed a sidewall gate structure using a work function-matched SWCNT channel and metal source and drain electrode materials, which solves the above problems and has good scaling characteristics and the ability to suppress the off-state current.

## 2. Structure, Modeling, and Operation Principle of Self-Aligned Gate CNTFET

The polarity of a CNTFET, whether n-type or p-type, is primarily governed by the work function of the metal contact interfacing with the CNT channel. To achieve p-type behavior in a CNTFET, a contact material with a higher work function than the CNT is used to promote hole injection, while n-type conduction is realized by employing a contact with a lower work function to facilitate electron injection. We chose palladium (Pd) as the electrode of CNT PMOS, which can make the metal–semiconductor contact an ohmic contact, and holes can pass through the channel and electrode without a barrier. Similarly, scandium (Sc) is selected as the electrode of CNTNMOS to ensure electrons can pass through the channel and electrode without a barrier. Sentaurus TCAD software (vO-2018.06) is employed in this work to model the CNTFET device.

In this study, carrier transport within CNTFETs was modeled using the classical drift–diffusion (DD) framework. To accurately represent mobility degradation resulting from carrier scattering mechanisms, the CarrierCarrierScattering model was incorporated. Additionally, we took advantage of the nonlocal band-to-band tunneling (BTBT) model to capture the BTBT currents occurring on the drain side in the off-state of the device. For precise characterization of the mobility–field relationship under drift–diffusion transport, the Caughey–Thomas formulation was utilized to ensure a continuous transition from a low-electric field to a high-electric field. The mobility is expressed as follows [28]:

(1) $$\;\mu_{n,p}=\mu_{0n,p}[\frac{1}{1+(\frac{\mu_{0n,p}E}{v_{satn,p}}{)}^{\beta }}{]}^{\frac{1}{\beta }}$$

*μ*_0*n*,*p*_ corresponds to the carrier mobility under low electric field conditions for electrons and holes. *E* corresponds to the component of the electric field parallel to the transport direction. *v_satn_*_,*p*_ indicates the saturation velocity of electrons and holes in high-field conditions, and *β* is a semi-empirical constant typically set to 1.4 [33,34]. The nondegenerate carrier concentration *n* of semiconducting CNTs is determined by [35,36]

(2) $$\;n=2N_{C}\exp(\frac{E_{F}-E_{C}}{kT})$$

(3) $$N_{C}=g_{0}\gamma_{0}\frac{2E_{VH1}+kT}{4E_{VH2}}\approx \frac{g_{0}}{4}\sqrt{\pi kTE_{g}}$$

(4) $$\;E_{g}=0.85/d_{CNT}$$

**Figure 1:**
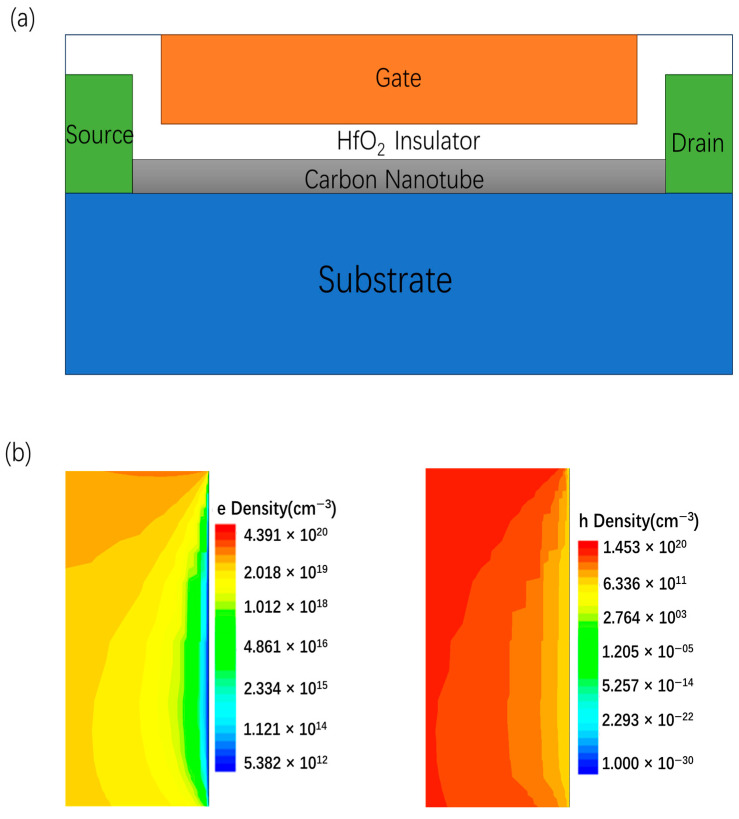
Self-aligned gate CNTFETs. Diagram showing the configuration of a self-aligned gate (**a**), on-state carrier concentration near the metal–semiconductor contact at drain side ((**b**) left-NMOS right-PMOS).

In which *N_C_* characterizes the effective number of states contributing to electron occupancy in the conduction band, *T* represents the temperature, *k* represents the Boltzman constant, *E_VH_*_1_ and *E_VH_*_2_ represent the energy levels corresponding to the van Hove singularities near the band extrema, *E_g_* represents the bandgap and *d_CNT_* is in nanometer [37], *g*_0_ represents the CNT material constant which is 2 nm^−1^·eV^−1^ [38], and *γ*_0_ represents the overlap energy between nearest neighbor atoms. *N_C_* is simplified by *E_VH_*_1_ + *kT*/2 ≈ *E_g_*/2 and *E_VH_*_2_ ≈ 2*γ* [35,36], as shown in Equation (3). It is calculated that the effective mass of CNT [33] is

(5) $$\;m^{*}=\frac{4\hbar^{2}E_{g}}{3\gamma a^{2}\left(2\gamma +E_{g}\right)}$$

where ℏ denotes the reduced plank constant, *γ* represents the nearest neighbor overlap energy (3.1 eV), and a represents the lattice constant (2.46 Å). Model calibration was performed based on experimental data reported in Ref. [29]. CNT FET with the self-aligned gate is shown in Figure 1a. Table 1 summarizes the key model parameters and geometrical dimensions used in the simulation.

The reason why we chose 2.4 nm diameter SWCNT is that it is in the middle of the large diameter range of SWCNT and has high on-current characteristics, but because the larger the diameter, the smaller the band gap, they usually have characteristics such as bipolarity, obvious off-state leakage, and limited current switching ratio. The research on 2.4 nm diameter devices is of great significance for improving large diameter devices.

Reasons for selecting the parameters N_C_ and N_V_ are as follows: for Nc, we refer to the experimental data [29] to locate the Nc value of CNTNMOS and CNTPMOS at 10^15^ cm^−3^ and 10^11^ cm^−3^, respectively, which can fit the on-state current and off-state current of the experimental results, respectively. For Nv, taking 10^15^ cm^−3^ in both CNTNMOS and CNTPMOS can fit the off-state current and on-state current in the experiment, respectively, which simplifies the simulation without affecting the accuracy of the results.

**Figure 2:**
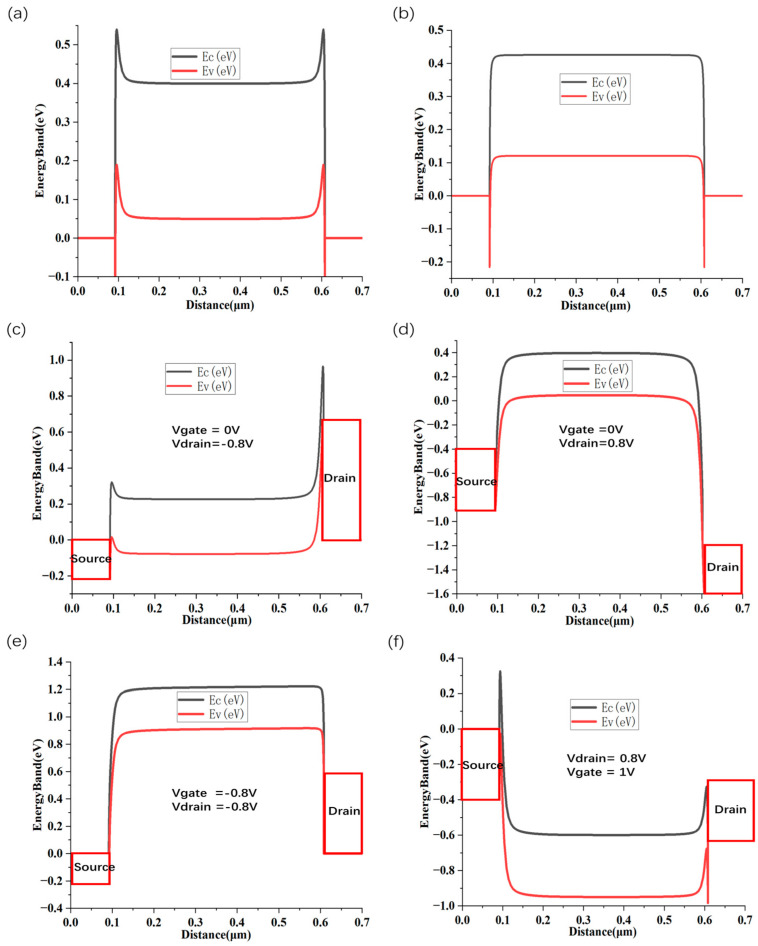
Energy band diagrams of CNTPMOS and CNTNMOS devices in different states. Energy band diagrams of p-type CNTFETs with ohmic contacts in thermal equilibrium (**a**), off state (**c**), and on state (**e**) and energy band diagrams of n-type CNTFETs in thermal equilibrium (**b**), off state (**d**), and on state (**f**).

In CNT FETs, the contact properties of p-type and n-type devices are determined by the metal’s work function. Due to the influence of metal–semiconductor contact, the majority carriers at the semiconductor interface will decrease rapidly as the distance from the electrode approaches. The carrier concentration at the on-state interface of NMOSFET and PMOSFET is shown (Figure 1b). At thermal equilibrium, the higher work function of the metal compared to the semiconductor leads to the formation of an energy barrier at the interface, affecting carrier injection. Upon contact, the semiconductor’s Fermi level shifts downward as electrons transfer from the semiconductor to the metal and holes are injected into the semiconductor, resulting in p-type behavior and the formation of an ohmic contact near the valence band. (Figure 2a). Similarly, when the metal has a lower work function than the semiconductor, an interfacial energy barrier is established, affecting carrier injection. In this case, a significant number of electrons are injected into the semiconductor, shifting its conductivity toward n-type and forming an ohmic contact aligned with the conduction band (Figure 2b).

**Figure 3:**
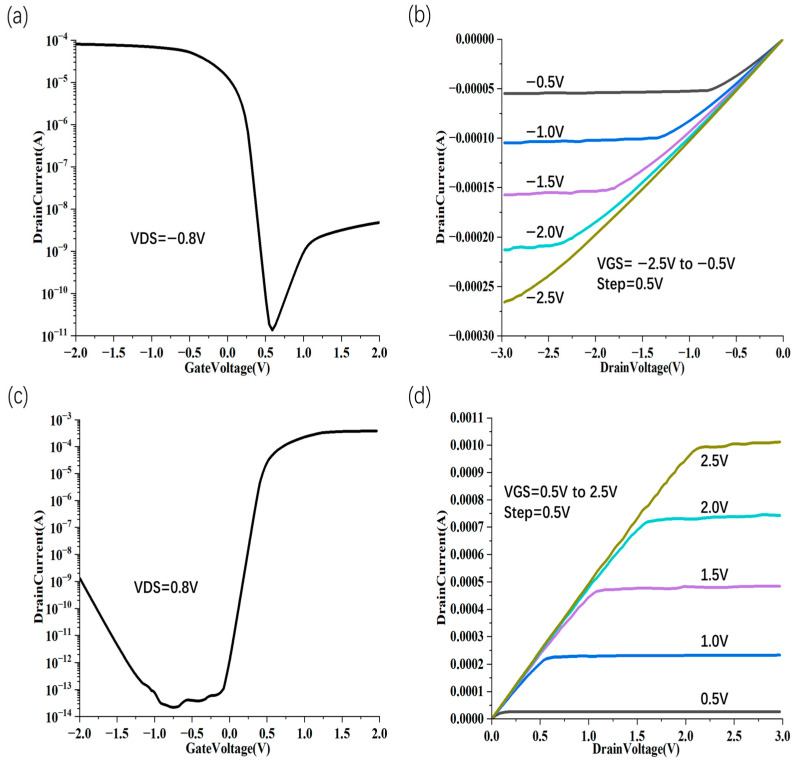
Transfer characteristic curves of self-aligned gate CNTFET. I_d_-V_g_ (**a**) and I_d_-V_d_ (**b**) transfer curves of CNTPMOS at different V_gs_. I_d_-V_g_ (**c**) and I_d_-V_d_ (**d**) transfer curves of CNTNMOS at different V_gs_.

When CNT PMOS is in the off-state (|V_gs_| < |Vth|) with a large bias (Figure 2c), holes are transmitted at the source–drain ohmic contact point, but face a large Schottky barrier on the drain side, so the hole current is very small. At this time (V_Source_ = 0 V, V_Gate_ = 0 V, V_Drain_ = −0.8 V), the electron Schottky barrier on the source side changes dramatically, the drain bias is large, the energy band per unit distance changes greatly, and a triangular barrier is formed near the drain area, resulting in barrier tunneling. At the same time, BTBT of electrons still exists, which will cause the off-state current of the device (Figure 3a) to be 10^−8^ A (electron band-to-band tunneling from right to left, current from left to right). Similarly, when CNT NMOS is in the off-state (|V_gs_| < |Vth|) with a large bias (Figure 2d), V_DS_ = 0.8 V, a triangular barrier is formed near the drain region, resulting in barrier tunneling. At the same time, BTBT of holes still exists, which causes the off-state current of the device (Figure 3c) to change from a minimum of 10^−13^ A to 10^−9^ A.

Under a negative gate bias in the CNT PMOS on-state condition (Figure 2e), the channel energy bands shift upward, significantly enhancing hole density while suppressing electron concentration. Via thermionic emissions, holes are able to surmount the potential barrier at the drain region and are efficiently injected into the drain, owing to the low-resistance ohmic contact, which leads to an open-state current (Figure 3a) of 10^−4^ A. When a positive voltage is applied to the CNT NMOS gate and the circuit is in the open state (Figure 2f), the channel energy band rises, the electron concentration is extremely high, and the hole concentration is extremely low. The electrons can cross the potential barrier near the drain by thermal electron emission and enter the drain with less hindrance in ohmic contact, which leads to an open-state current (Figure 3c) of 4 × 10^−4^ A. A significant saturation current, I_sat_, is observed in the device, about 250 µA for PMOS (Figure 3b), and about 10 mA for NMOS (Figure 3d). The subthreshold swing SS of this MOSFET (diameter d = 2.4 nm) is about 70 mV/decade (Figure 3a).

Based on the above discussion, the SAG structure CNT FET faces the problem of an excessive off-state current that needs to be solved in both n-type and p-type.

## 3. Proposal of Scaling Structure of CNTFETs and Reduction in Leakage Current

### 3.1. Vertically Scaling Structure

The preceding analysis of leakage factors suggests that to suppress bipolar conduction in CNT FETs, it is effective to reduce the gate’s electric field influence near the drain side and simultaneously enhance the drain-induced field at the contact region, which helps to suppress the formation of a triangular potential barrier. Therefore, we need an appropriate gate oxide thickness (L_spacer_) and an increase in the electric field effect of the drain region on the drain side of the semiconductor (L_ext_). To further reduce the device footprint, it is also necessary to strengthen the modulation of the drain-side band by the drain potential. Accordingly, we introduce a vertically aligned CNT FET architecture, in which the source and drain electrodes are vertically stacked.

**Figure 4:**
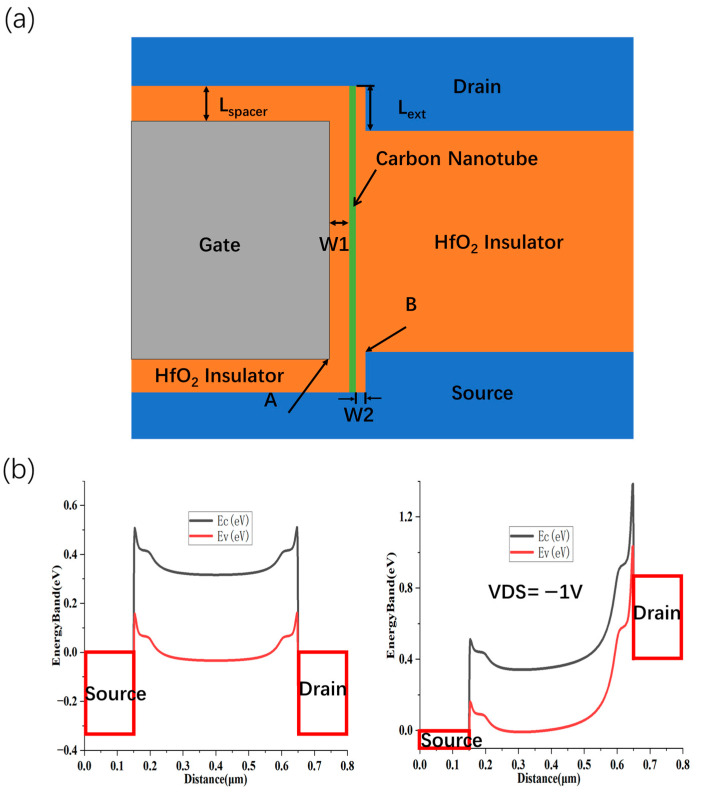
Source–drain vertically stacked CNT FET. CNT FET structure of vertically stacked structure (**a**), channel energy band diagram under thermal equilibrium state and V_DS_ = −1 V state (**b**).

Table 2 lists the required model parameters and geometric dimensions. Other parameters are the same as the previous. A schematic of the vertically stacked CNT FET is presented (Figure 4a). Relative to planar transistor architectures, it offers two distinct advantages.

Firstly, the transport channel in the proposed architecture is oriented vertically, orthogonal to the substrate plane, with its effective length defined by the dielectric spacer thickness between the source and drain terminals. Special attention is given to the bottom source contact, which is deliberately engineered to be relatively thick. This design choice ensures robust electrostatic gate control over the proximal segment of the SWCNT channel. In scenarios where the height of the bottom source electrode is insufficient, the gate dielectric deposited on the raised source feature may cause the lower edge of the vertical gate electrode (point A) to exceed the top surface of the source electrode (point B). Such a misalignment compromises gate-channel capacitive coupling and leads to electrostatic underlap, thereby reducing gate modulation efficiency in this critical region.

**Figure 5:**
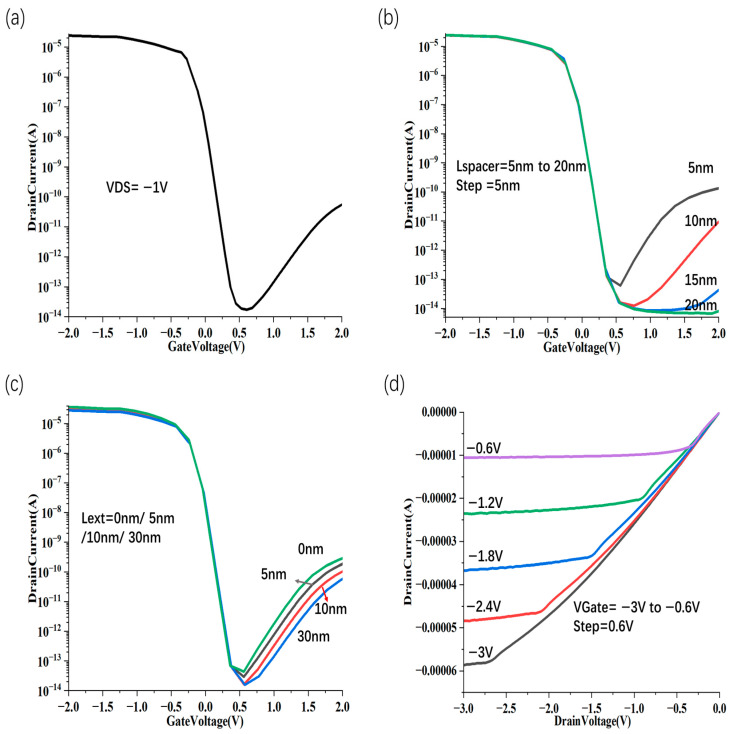
I_d_-V_g_ transfer characteristic curve diagram (**a**), transfer characteristic curve of vertically stacked CNT FET with different L_spacer_ when W_1_ = W_2_ = 8 nm, L_ext_ = 50 nm (**b**), transfer characteristic curve of vertically stacked CNT FET with different L_ext_ when W_1_ = W_2_ = 8 nm, L_spacer_ = 8 nm (**c**) and I_d_-V_d_ transfer characteristic curve simulation diagram in L_g_ = 400 nm (**d**), the gate voltage changes from −3.0 V to −0.6 V.

Secondly, in contrast to conventional device configurations, the conduction and valence bands of the CNT channel in the proposed structure are more effectively aligned with the drain potential, forming a broader energy barrier at the drain electrode, which is depicted in Figure 4b. This enlarged tunneling barrier significantly impedes hole injection from the drain reservoir, thus offering effective mitigation of gate-induced drain leakage (GIDL). At V_DS_ = –1 V, the vertically stacked device demonstrates an off-state leakage current (I_off_) approximately 100 times lower than that of the self-aligned gate structure, as illustrated in Figure 5a. We also scale the spacer thickness L_spacer_ (Figure 5b). The results show that due to the small bandgap width of CNTS with a large diameter, the device I_off_ is very sensitive to L_spa_, and when L_spa_ is less than 5 nm, the device I_off_ is greater than 10 pA/µm. In order to make the device have a smaller off-state current, L_spa_ should be less than or equal to 10 nm.

**Figure 6:**
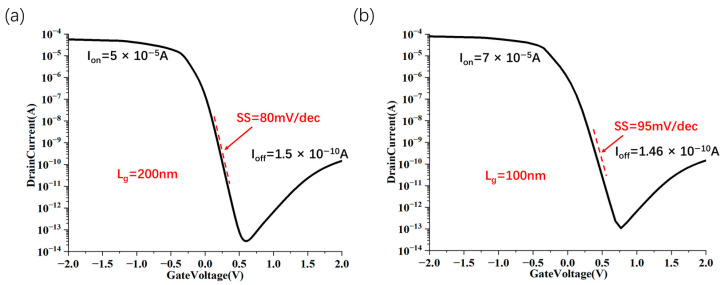

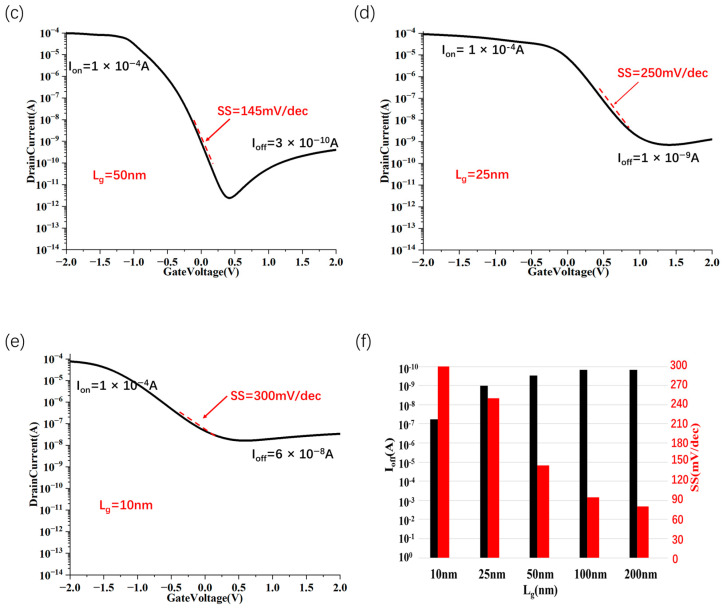
Scaling characteristics of source–drain vertically stacked CNT FET. Scaling behavior of gate length from 10 nm to 200 nm of source–drain stacked CNT FET on on/off ratio and SS index at an operating voltage of −1 V (**a**–**e**), the L_spa_ of the corresponding device is 10 nm and L_ext_ is 10 nm. Summary of the gate length scaling behavior in the on/off ratio and SS metrics of the source–drain stacked CNT FET at an operating voltage of −1 V (**f**).

As shown in Figure 5c, for different lengths of the horizontal isolation layer (L_ext_), when L_ext_ is reduced to between 10 nm and 30 nm, the circuit has a better leakage current suppression effect. The I_d_-V_g_ transfer characteristic curve of the device is shown in Figure 5d, which reflects the effective control of the gate and drain over the on-state current. Finally, to investigate scalability, we computed the transfer characteristics for gate lengths (L_g_) varying from 10 to 200 nm. Figure 6 extracts the on/off current and SS of CNT FETs with different L_g_ stacking structures. Among them, the devices with gate lengths of 200 nm, 100 nm, 50 nm and 25 nm have good electrical characteristics (on/off ratios are 3.3 × 10^5^, 4.8 × 10^5^, 3.3 × 10^5^, 1 × 10^5^, respectively; SS are 80 mV/dec, 95 mV/dec, 145 mV/dec, 250 mV/dec, respectively). Owing to the intensified short channel effects, at a gate length of 10 nm, the subthreshold swing (SS) reaches only 300 mV/dec, and the on/off current ratio is 1.6 × 10^3^. Consequently, when the gate length is reduced to 10 nm or less (with a channel length of 30 nm), the device structure ceases to function effectively.

**Figure 7:**
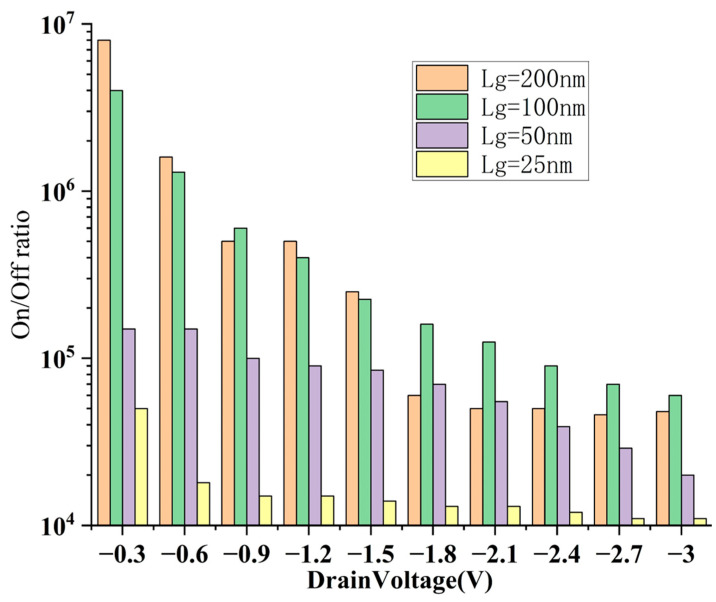
Stability of on/off ratio at different V_DS_ and L_g_ of vertically stacked structure.

We also simulate and analyze the stability of the on/off ratio of this structure under different V_DS_ (Figure 7). For the vertically stacked structure, within the range of V_DS_ = 0∼−3 V, the on/off ratio variation in both long channel and short channel devices is controlled within about two orders of magnitude, indicating that the structure has good electrical stability over a wide voltage operating range. In particular, in the short channel scenario, the on/off ratio fluctuates less with the change in V_DS_, showing better SCE resistance than the long channel. This is attributed to the enhanced gate-controlled electric field wrapping characteristics in the vertically stacked structure, which effectively suppresses the occurrence of DIBL effect, thereby maintaining a low off current I_off_.

**Figure 8:**
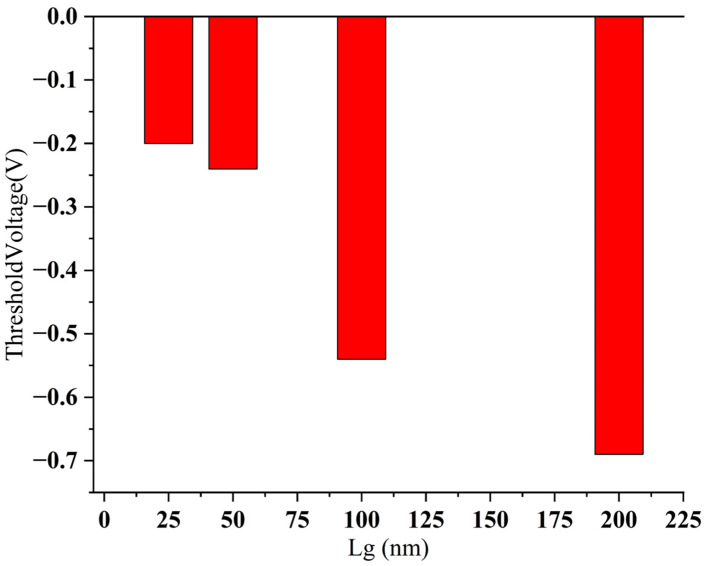
Behavior of threshold voltage at different L_g_ of vertically stacked structure.

We also discuss the threshold voltage behavior with scaling (Figure 8). For the vertically stacked structure (PMOS), the simulation results show that its threshold voltage gradually increases (i.e., the absolute value decreases) as the channel length shortens, and the specific value increases from −0.69 V (long channel) to −0.20 V (short channel), showing a clear threshold voltage roll-up trend. To conclude, the sidewall gate structure exhibits obvious threshold voltage roll-off under channel scaling conditions.

**Figure 9:**
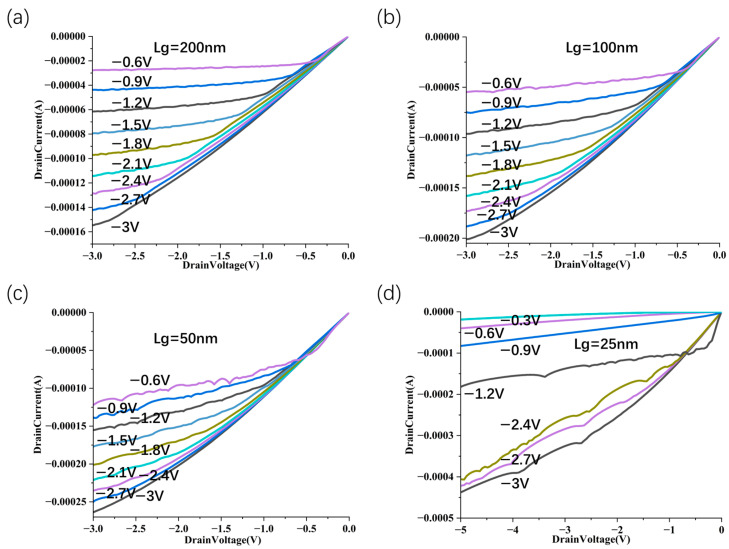
Channel length modulation effect of vertically stacked structure. I_D_-V_D_ performance with different channel lengths: L_g_ = 200 nm (**a**), L_g_ = 100 nm (**b**) L_g_ = 50 nm (**c**) L_g_ = 25 nm (**d**) with the gate voltage changing from −3.0 V to −0.6 V.

We performed simulation analysis on SCE parameters such as DIBL, threshold voltage roll-off, and channel length modulation. First, the threshold voltage roll-offs for the vertically stacked structures are as follows: 200 nm–100 nm is 1.3 mV/nm, 100 nm–50 nm is 5 mV /nm, and 50 nm–25 nm is 3.2 mV/nm (Figure 8). From the results, it is clear that in the long channel, the gate has a strong control over the channel potential, the short channel effect is weak, and the threshold voltage does not change much. Under the condition of a medium-length channel, the short channel effect begins to increase significantly. After further shrinking to an ultra-short channel (25 nm), the threshold voltage still decreases, but the roll-off amplitude is moderated. Second, for DIBL parameters, the vertically stacked structure has a L_g_ of 200 nm corresponding to 35 mV/V, a L_g_ of 100 nm corresponding to 70 mV/V, a L_g_ of 50 nm corresponding to 150 mV/V, and a L_g_ of 25 nm corresponding to 230 mV/V. As the channel length decreases, the DIBL value rises sharply from 35 mV/V to 230 mV/V, showing that the barrier height of the short channel device is significantly reduced under electric field interference, reflecting a stronger SCE. Third, for the channel length modulation parameters (Figure 9), L_g_ = 200 nm corresponds to 0.08 V^−1^, L_g_ = 100 nm corresponds to 0.21 V^−1^, L_g_ = 50 nm is 0.22 V^−1^ when the drain voltage is low and 0.3 V^−1^ when the drain voltage is high, and L_g_ = 25 nm is 0.26 V^−1^ when the drain voltage is low and 0.35 V^−1^ when the drain voltage is high. This structure has good performance in the medium and long channels but an average CLM performance in the short channel region.

**Figure 10:**
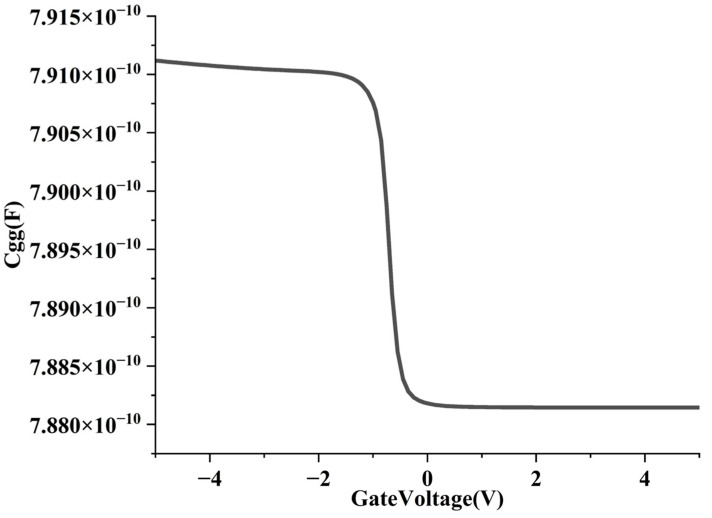
CV characteristics of vertically stacked structure.

We analyze the CV characteristics (Figure 10). The C_gg_ (total gate capacitance) curve trend of PMOS is as follows: the C_gg_ is maintained at 7.91 × 10^−10^ F when the gate voltage increases from −5 V to −1 V, rapidly decreases to 7.88 × 10^−10^ F when the gate voltage increases from −1 V to 0 V, and remains unchanged when the gate voltage increases from 0 V to 5 V.

Impact on switching speed: gate delay τ = R × C_gg_, in which R is the drive resistance and C_gg_ is the total gate capacitance. In the −5 V to −1 V range, PMOS is in a saturated conduction state, with sufficient carriers, stable capacitance, and predictable switching speed; in the −1 V to 0 V range, the capacitance drops rapidly, indicating that the channel is in a depleted state and the switching speed becomes faster. The device has a sudden drop in capacitance when it is close to the cutoff point (near −1 V), which is conducive to increasing the turn-off speed and reducing the switching time.

Impact on energy delay product (EDP): EDP = E × τ = 0.5 × C_gg_ × Vdd^2^ × τ. It can be seen that C_gg_ has a secondary effect on EDP (affecting both power consumption and delay). In the on state, C_gg_ is stable, and E and τ are controlled by it at the same time; in the turn-off transition region, the capacitance decreases, that is, energy consumption is reduced (E∝C), and the delay is reduced (τ∝C). Therefore, the steep drop in C_gg_ helps to significantly reduce EDP and improve energy efficiency.

The results above show that the structure has good SS, DIBL, and channel length modulation effect parameters, as well as a good threshold voltage roll-off in medium-length channels. In extremely short channels, the channel length modulation effect is better, and SS, DIBL, and threshold voltage roll-off deteriorate to a certain extent, but these parameters are close to the average level.

### 3.2. Scaling Structure with sub-1 nm Sidewall Gate Length

In the realm of 2D-material-based transistors, three primary gate architectures are commonly employed: top-gated, embedded-gate, and bottom-gated configurations. Regardless of whether a global or localized gating scheme is adopted, the physical gate length (L_g_) is generally constrained by the inherent resolution limits of lithographic techniques. Even with advanced patterning methods such as electron beam lithography (EBL), achieving gate lengths below 5 nm remains highly challenging [39]. Technologies to shorten the effective channel length to the nanometer level have been proposed, which provides us with inspiration for device scaling [40,41].

The previous section solved the problem of suppressing leakage current in medium and long-channel MOSFETs, but for short-channel devices, the off-state current suppression effect is poor. To overcome this limitation, a new device architecture is introduced, allowing gate length scaling through selective gating of the vertical transport channel. At the same time, this structure weakens the tunneling phenomenon caused by the large gate-drain potential difference in the above two parts by non-horizontal contact between source and drain electrodes and the channel.

**Figure 11:**
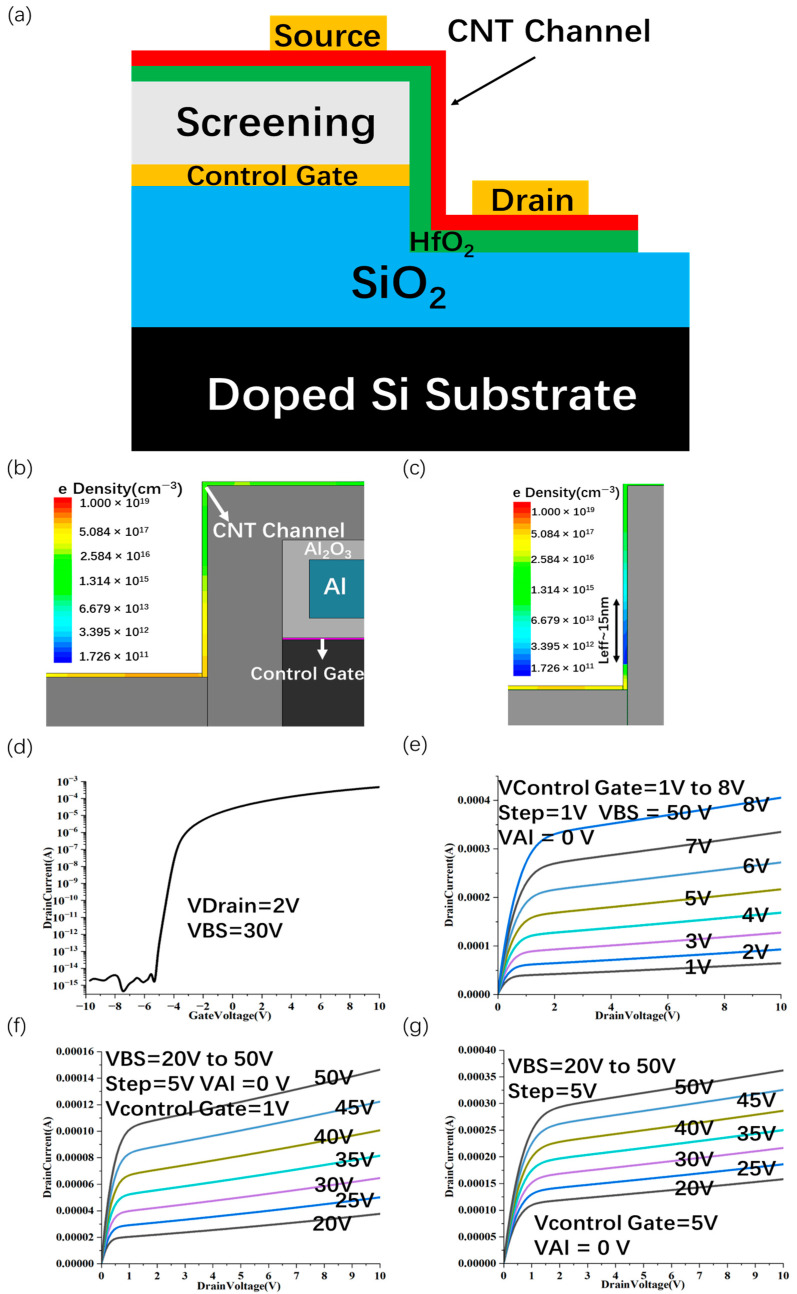
Sidewall gate CNT FET. Sidewall gate CNT FET with sub-1 nm gate length (**a**) and channel carrier concentration at on-state V_DS_ = 2 V, V_control gate_  =  5 V, V_BS_  =  30 V, V_Al_  =  0 V (**b**) and off-state V_DS_  =  2 V, V_control gate_  =  −4 V, V_BS_  =  30 V, V_Al_  =  0 V (**c**). I_d_-V_d_ transfer characteristic curve (**d**) and I_d_-V_d_ transfer characteristic curve (**e**) at V_BS_ = 50 V and V_Al_ = 0 V, control gate voltage changes from 1 V to 8 V with 1 V interval. I_d_-V_d_ transfer characteristic curve at control gate voltage of 1 V (**f**) and 5 V (**g**), substrate voltage changes from 20 V to 50 V with 5 V interval.

Planar device architectures with coplanar source, drain, and gate show substantial leakage when the gate length is scaled to the nanometer regime, as evidenced by the previously discussed energy band diagrams. Under elevated drain bias conditions, pronounced tunneling currents are likely to occur at the drain junction. To address these limitations, we introduce an optimized device configuration: a sidewall two-dimensional (2D) transistor, depicted in Figure 11a. This novel design utilizes the gate electrode edge to define an ultra-short gate length of less than 1 nm, enabling precise electrostatic control over the carbon nanotube (CNT) channel. Furthermore, an additional aluminum (Al) layer serves as an electrostatic shield, attenuating the vertical electric field emanating from the Al surface, thereby confining the effective gate field to originate exclusively from the gate edge. Consequently, only a segment of the vertical CNT channel is modulated by the gate potential. The detailed device parameters and geometric specifications are provided in Table 3.

In the sidewall-gated CNT transistor architecture, three distinct terminals are capable of modulating the carrier concentration within the channel: (1) the primary control gate; (2) the aluminum shielding electrode; (3) the silicon back-gate terminal. During simulation, only the control gate is actively biased, while the Si back-gate is maintained at a constant potential of 30 V and the Al shielding layer is grounded (0 V). This configuration effectively screens the vertical electric field over the top surface of the control gate, thereby allowing the fringe field at the gate edge to solely influence the vertical CNT channel. As a result, an ultra-short effective gate length of approximately 0.44 nm is achieved.

We control the on and off state of the channel by changing the control gate voltage. The electron density along the vertical CNT channel in the on state (V_control gate_ = 5 V) and the off state (V_control gate_ = −4 V) is also shown under V_DS_ = 2 V, V_BS_ = 30 V, and V_Al_ = 0 V (Figure 11b,c). The control gate is gated by directly controlling the channel carrier concentration near the gate by changing its voltage. By considering the effective channel length (L_eff_) as the region where the carrier concentration remains below a threshold value of n < n_TH_ = 3 × 10^12^ cm^−3^, the segment of the CNT sidewall approximately 15 nm adjacent to the control gate interface can be identified as the active transport channel. The Sc-contacted SWCNT FET exhibits metallic behavior in n-channel conduction (Figure 11d) and highly linear conductance at high V_ds_ (Figure 11e). In addition, the effect of substrate voltage on channel conduction is similar to that of the control gate.

A significant increase in substrate voltage can affect the on-current (Figure 11f,g), but its effect is weaker than that of the control gate.

**Figure 12:**
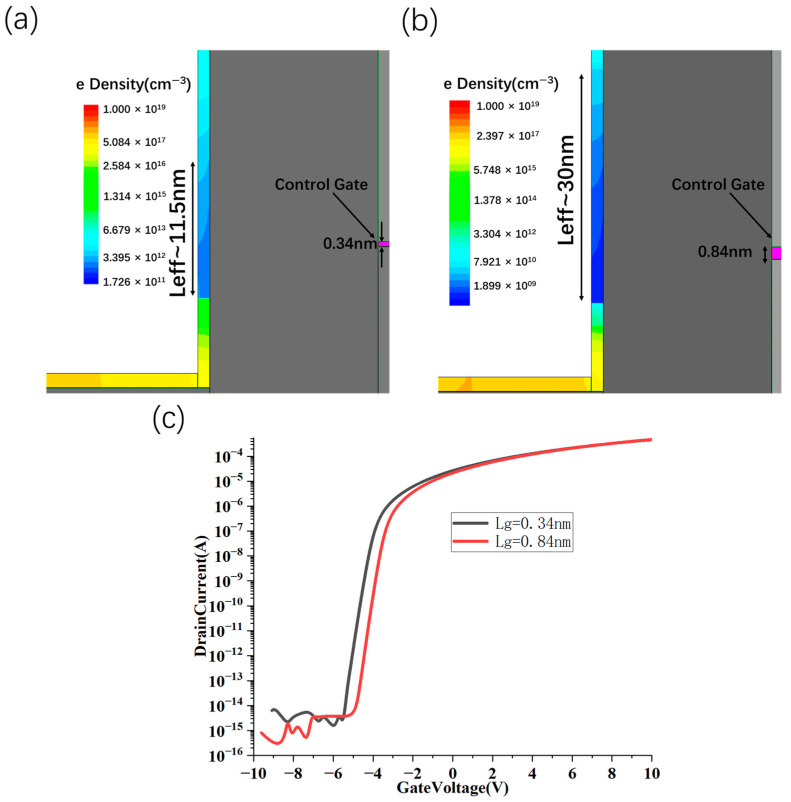
Scaling characteristics and transfer characteristics of sidewall gate CNT FET. The equivalent channel length of the sidewall gate CNT FET with gate length of 0.34 nm (**a**) and 0.84 nm (**b**) is 11.5 nm and 30 nm, respectively, when V_DS_ = 2 V, V_control gate_ = −4 V and V_BS_ = 30 V in the off state. Transfer characteristics curve when V_control gate_ = 2 V, V_BS_ = 30 V (**c**).

To explore the scalability of the device, we calculated the transfer characteristics of the gate length in the sub-1 nm range (Figure 12). Among them, the devices with gate lengths of 0.84 nm and 0.34 nm have good electrical characteristics: the on/off ratio is 5 × 10^10^ and 6.3 × 10^10^, respectively; SS is 200 mV/dec and 250 mV/dec, respectively. Among them, the 0.84 nm gate length structure can obtain an effective channel length of 30 nm, which effectively suppresses the problem of excessive device leakage when the L_g_ is 10 nm (effective channel length is 30 nm) in the previous part.

**Figure 13:**
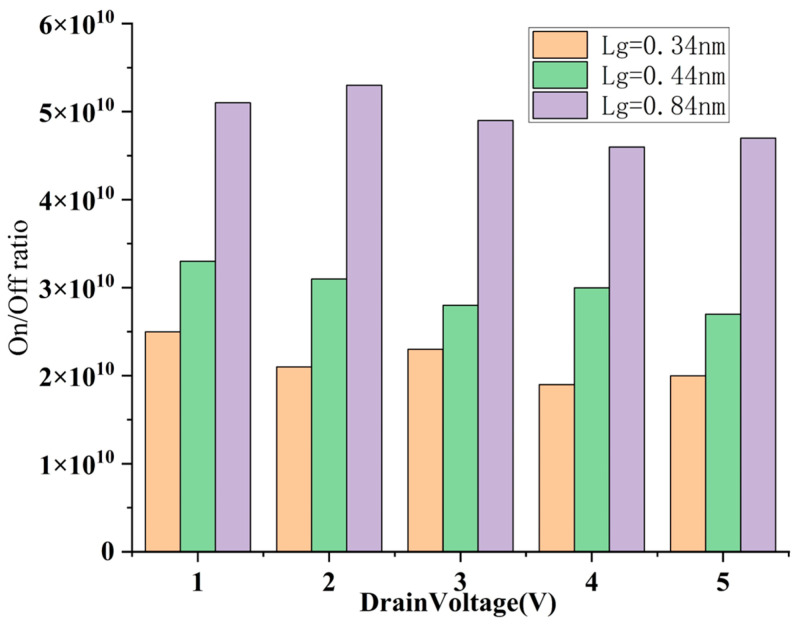
Stability of on/off ratio at different V_DS_ and L_g_ of sidewall gate CNT FET.

We simulate and analyze the stability of the on/off ratio of this structure under different V_DS_ (Figure 13). For the sidewall gate structure, the simulation results show that it maintains a high switching ratio stability at different channel lengths, and the on/off ratio changes slowly with V_DS_, with the range of change controlled within about one order of magnitude. This shows that the sidewall gate structure also has good gate control capabilities at the nanoscale, and can effectively alleviate the problems of threshold voltage roll-off and leakage current increase caused by short channels, and is particularly suitable for integration scenarios that are sensitive to energy consumption or operate at low voltage.

**Figure 14:**
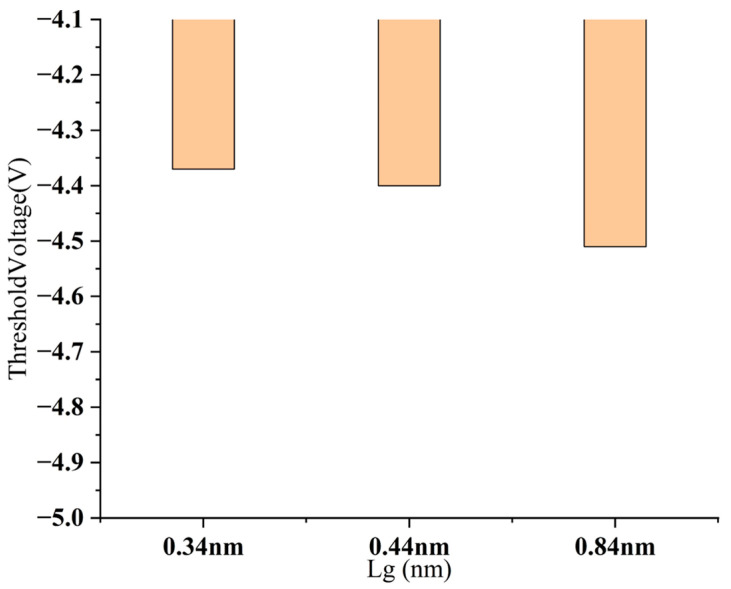
Behavior of threshold voltage at different L_g_ of sidewall gate CNT FET.

We discuss the change in threshold voltage with scaling (Figure 14). For the sidewall gate structure (NMOS), the threshold voltage increases as the channel length decreases, from −4.51 V to −4.37 V. To conclude, the sidewall gate structure exhibits obvious threshold voltage roll-off under channel scaling conditions.

**Figure 15:**
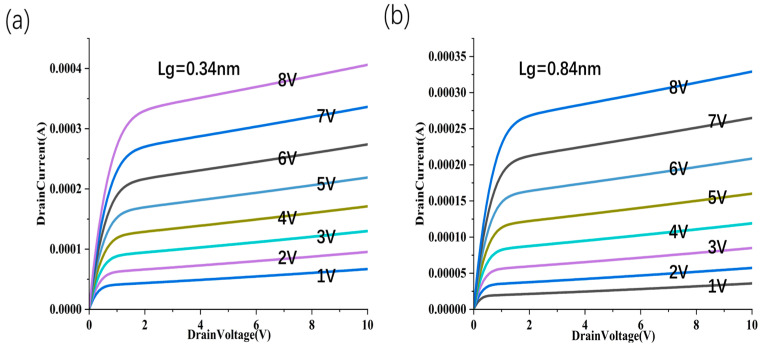
Channel length modulation effect of sidewall gate CNT FET. I_D_-V_D_ performance with different channel lengths: L_g_ = 0.34 nm (**a**), L_g_ = 0.84 nm (**b**) with the gate voltage changing from 1 V to 8 V.

We performed simulation analysis on SCE parameters such as DIBL, threshold voltage roll-off, and channel length modulation. (1) Threshold voltage roll-off, for the sidewall gate CNT FET, it has a 0.84 nm–0.44 nm value of 5.8 mV/nm, and 0.44 nm–0.34 nm corresponds to 0.6 mV/nm (Figure 14). Judging from the results, the sidewall gate structure still exhibits good Vth control capabilities at the sub-nanometer level, especially in the shortest channel range (0.44–0.34 nm), where there is almost no significant roll-off, reflecting extremely strong electric field shielding and short channel control capabilities. (2) DIBL parameters. The sidewall gate structure has a gate length of 0.84 nm corresponding to 90 mV/V, 0.44 nm corresponding to 113 mV/V, and 0.34 nm corresponding to 130 mV/V. Despite being in the ultra-short channel range, the sidewall gate structure still exhibits a relatively controllable DIBL value (<150 mV/V), which is better than the vertically stacked structure of the same size. (3) Channel length modulation parameters (Figure 15). The sidewall gate structure has a 0.84–0.44 nm value of 5.8 mV/nm, and 0.44–0.34 nm corresponds to 0.6 mV/nm. Judging from the results, the sidewall gate structure still exhibits good V_TH_ control capabilities at the sub-nanometer level, especially in the shortest channel range (0.44–0.34 nm), where there is almost no significant roll-off, reflecting extremely strong electric field shielding and short channel control capabilities.

**Figure 16:**
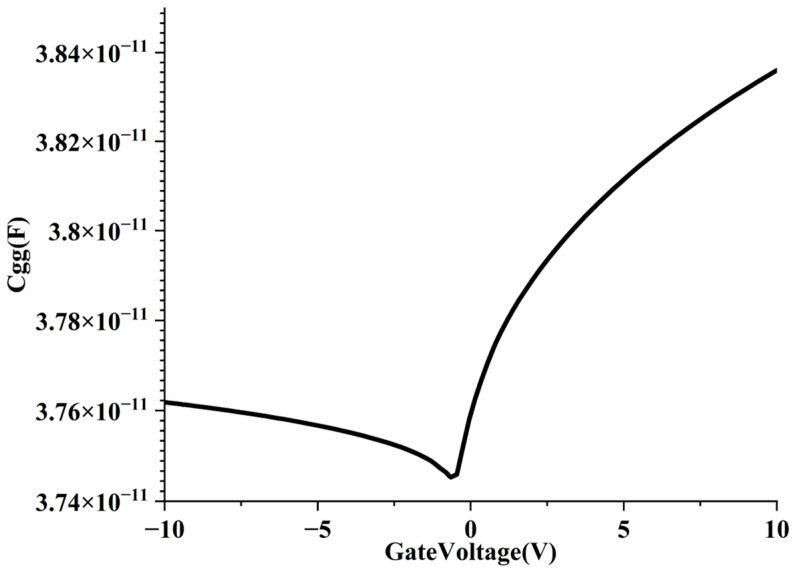
CV characteristics of sidewall gate CNT FET.

We analyze the CV characteristics (Figure 16). For the sidewall gate CNT FET, the trend of the C_gg_ curve of NMOS is as follows: C_gg_ slowly decreases from 3.76 × 10^−11^ F to 3.745 × 10^−11^ F, when the gate voltage increases from −10 V to 0 V, and rapidly increases to 3.84 × 10^−11^ F when the gate voltage increases from 0 V to 10 V. The overall trend shows that the NMOS device has good gate capacitance controllability, especially the capacitance response is fast near the switching point.

Impact on switching speed: between −10 V and 0 V: C_gg_ changes very slowly and is almost constant. In this range, the device is in the off state, the dynamic response is weak, and the energy consumption is extremely small, but it will not participate in effective switching. Between 0 V and 10 V, the capacitance rises rapidly, from 3.745 × 10^−11^ F to 3.84 × 10^−11^ F; it will increase the gate delay, but it means that the device responds to input switching more quickly, which is conducive to logic flipping. The steep curve means that the device has a faster response speed.

Impact on energy-delay product (EDP): in the on-state region, the increase in C_gg_ also brings about an increase in energy consumption E and delay τ; but because the capacitance increase is limited (<3%), it will not cause a significant deterioration of EDP; at the same time, the fast capacitance response also reduces the uncertainty delay during the voltage flip, which is conducive to improving the energy efficiency ratio; in the off-state region, C_gg_ is almost unchanged, ensuring extremely low static power consumption.

The results show that the structure has good SS, DIBL, channel length modulation effect parameters, and relatively good threshold voltage roll-off. At the same time, its CV characteristics show that it has a good switching rate and good energy delay parameters.

It should be pointed out that purely relying on simulation methods still has certain limitations. The physical models and material parameters used in the simulation process are usually based on idealized assumptions, which cannot fully reflect the carbon nanotube structural defects, inter-tube coupling effects, contact interface uncertainties, and non-ideal factors introduced by the process that may exist in the actual device preparation. Moreover, the future directions of our work are as follows: in the field of digital circuits, this device structure is expected to be applied to low-power logic units, advanced scalable integrated platforms, AI edge computing circuits, and high-density SRAM and reconfigurable logic modules; in neuromorphic, it can be used in memory-computing units (in-memory computing), and the low leakage characteristics ensure the accuracy of the analog computing process; in RF circuits, it can be applied to many modules, such as switch matrices, modulators, and LNA control paths to reduce power consumption.

## 4. Conclusions

In this work, we first constructed a CNT FET model based on experimental data and TCAD simulation. Then, by analyzing the cause of its off-state current, we innovatively proposed a design strategy to extend the length of the source and drain epitaxial region (L_ext_) in the vertical stacking structure to enhance the drain electric field control and thus suppress the off-state current. This structure has good scaling characteristics and an off-state current suppression effect when L_g_ is between 200 nm and 25 nm. The off-state current of this structure (L_g_ = 25 nm) is 10^−9^ A, which is compared with Reference [23] (10^−6^ A), proving that this structure suppresses the off-state leakage current of short-channel devices by at least three orders of magnitude. In view of the failure of the vertical stacking structure to suppress the off-state current when L_g_ is 10 nm, we proposed a sidewall gate structure using a work function-matched SWCNT channel and metal source and drain electrode materials. This structure has good scaling ability and strong ability to suppress leakage current (on–off ratio greater than 10^10^). The off-state current (10^−14^ A) of this structure is reduced by at least eight orders of magnitude compared to the off-state current (5 × 10^−6^ A) in Reference [24]. Compared to Reference [39], the off-state current is reduced by one order of magnitude, and the on-state current is increased by one order of magnitude.

## Figures and Tables

**Table 1:** Model parameters of self-aligned gate CNT MOS device.

Parameters	Value
Diameter (*d_CNT_*) (nm)	2.4
Bandgap (*E_g_*) (eV)	0.35
CNT Affinity (eV)	4.4
Effective Mass (m*)	0.029
Mobility of CNT Channel (*μ*_0_)	1 × 10^4^
NMOS Effective DOS (*N_C_*) (cm^−3^)	3.4 × 10^15^
PMOS Effective DOS (*N_C_*) (cm^−3^)	3.4 × 10^11^
Effective DOS (*N_V_*) (cm^−3^)	6.65 × 10^15^
Saturation Velocity (*V_sat_*) (cm/s)	1.1 × 10^7^
NMOS Contact Workfunction (Φ_m_) (eV)	2.9
PMOS Contact Workfunction (Φ_m_) (eV)	5.1
Gate Workfunction of NMOS (Φ_G_) (eV)	4.8
Gate Workfunction of PMOS (Φ_G_) (eV)	5.1
Channel Length of CNT (nm)	516
Thickness of HfO_2_ (nm)	8

**Table 2:** Model parameters of vertically stacked CNT FET.

Parameters	Value
Effective DOS (*N_C_*) (cm^−3^)	3.4 × 10^11^
Effective DOS (*N_V_*) (cm^−3^)	6.65 × 10^16^
Contact Workfunction (Φ_m_) (eV)	5
Gate Workfunction (Φ_G_) (eV)	4.7
Channel Length of CNT (nm)	500
Gate Length (nm)	484
L_spacer_ (nm)	8
L_ext_ (nm)	50
W_1_ (nm)	8
W_2_ (nm)	8

**Table 3:** Model parameters of scaling structures with sub-1 nm sidewall gate length.

Parameters	Value
Effective DOS (N_C_) (cm^−3^)	3.4 × 10^14^
Effective DOS (N_v_) (cm^−3^)	6.65 × 10^13^
Contact work function (Φ_M_) (eV)	2.7
Gate work function (Φ_G_) (eV)	5.4
Length of Source and Drain (nm)	30
Thickness of Al_2_O_3_ (nm)	5
Length of Control Gate (nm)	200
Thickness of Control Gate (nm)	0.44
Channel Length of CNT (nm)	800
Thickness of CNT (nm)	2.4
Thickness of HfO_2_ (nm)	14
Thickness of Aluminum Layer (nm)	15
Thickness of Source side SiO_2_ (nm)	50
Thickness of Drain side SiO_2_ (nm)	26

## Data Availability

The original contributions presented in this study are included in the article. Further inquiries can be directed to the corresponding author.
